# Supplementary material for: Organization of high-level visual cortex in human infants
Source: Nat Commun. 2017 Jan 10;8:13995. doi: 10.1038/ncomms13995 (PMC5234071; doi:10.1038/ncomms13995)
Supplement: Supplementary Information — Supplementary Figures and Supplementary Tables [file ncomms13995-s1.pdf]

Supplementary Fig. 1: Sample frames from movie stimuli used in Expt. 2.

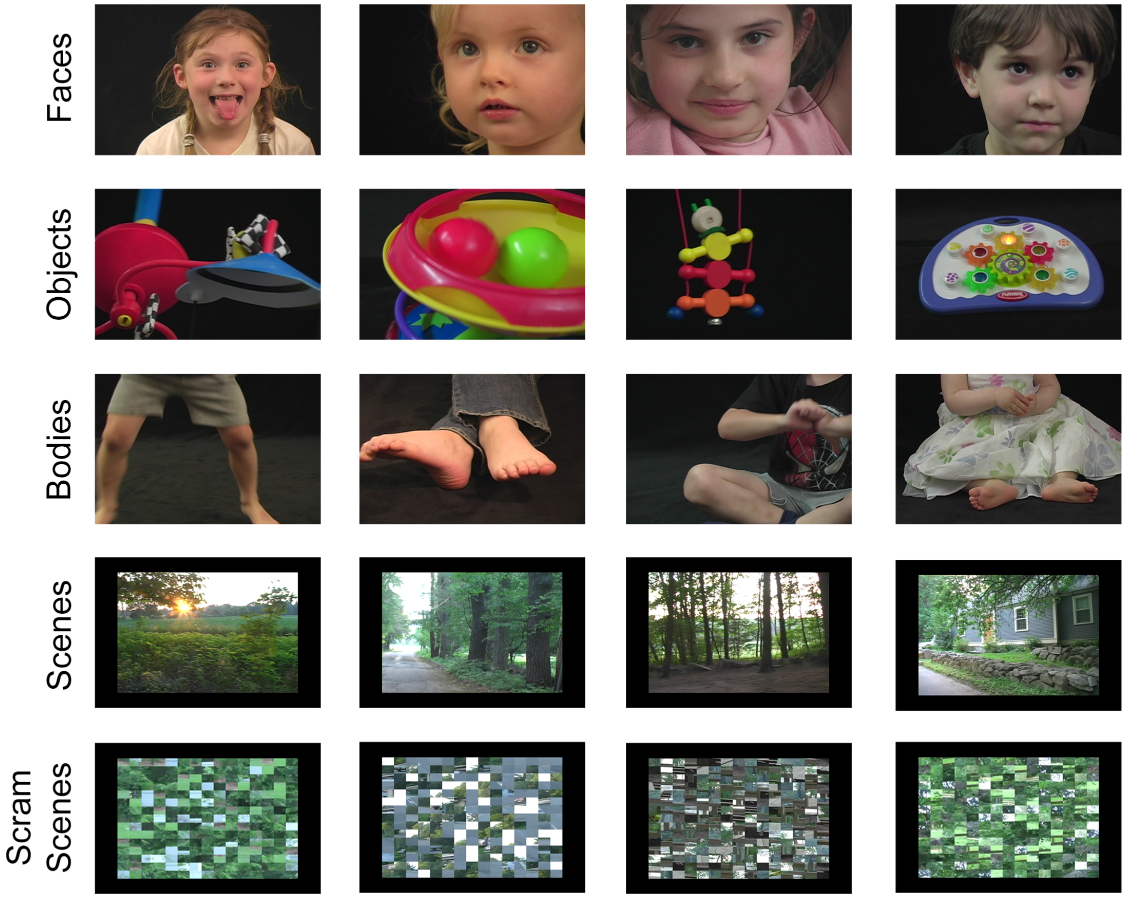

Supplementary Fig. 2: Whole-brain activation maps, comparing faces to scenes, in all infants and datasets not included in Fig. 1. Maps are thresholded at $P < .01$ voxelwise, and corrected for multiple comparisons using a clusterwise threshold of $P < .05$.

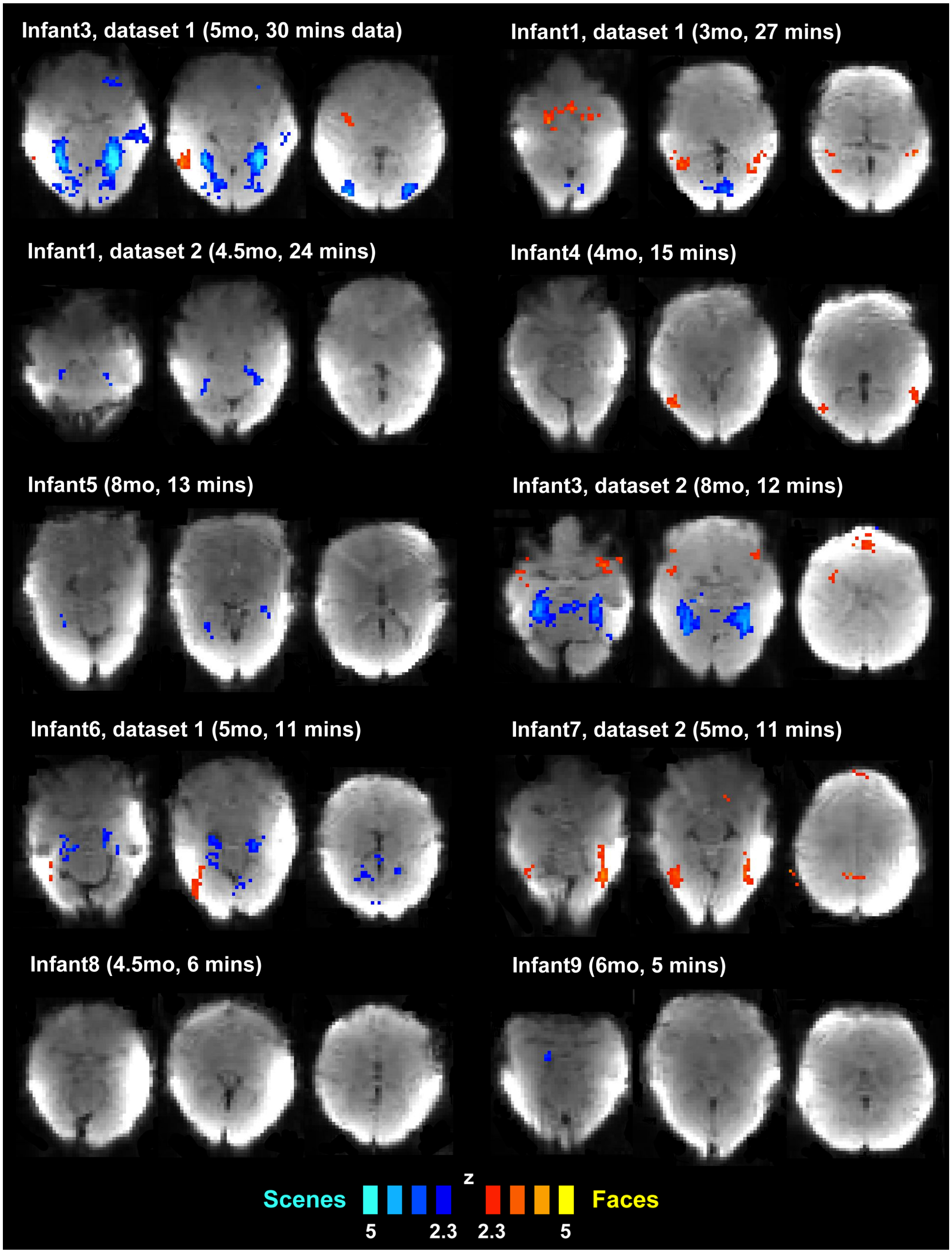

Supplementary Fig. 3: Whole-brain activation maps in a representative adult participant. The top row of images shows responses to faces versus scenes, while the bottom row shows comparisons of faces to objects and scenes to objects. Maps are thresholded at $P < .01$ voxelwise, and corrected for multiple comparisons using a clusterwise threshold of $P < .05$.

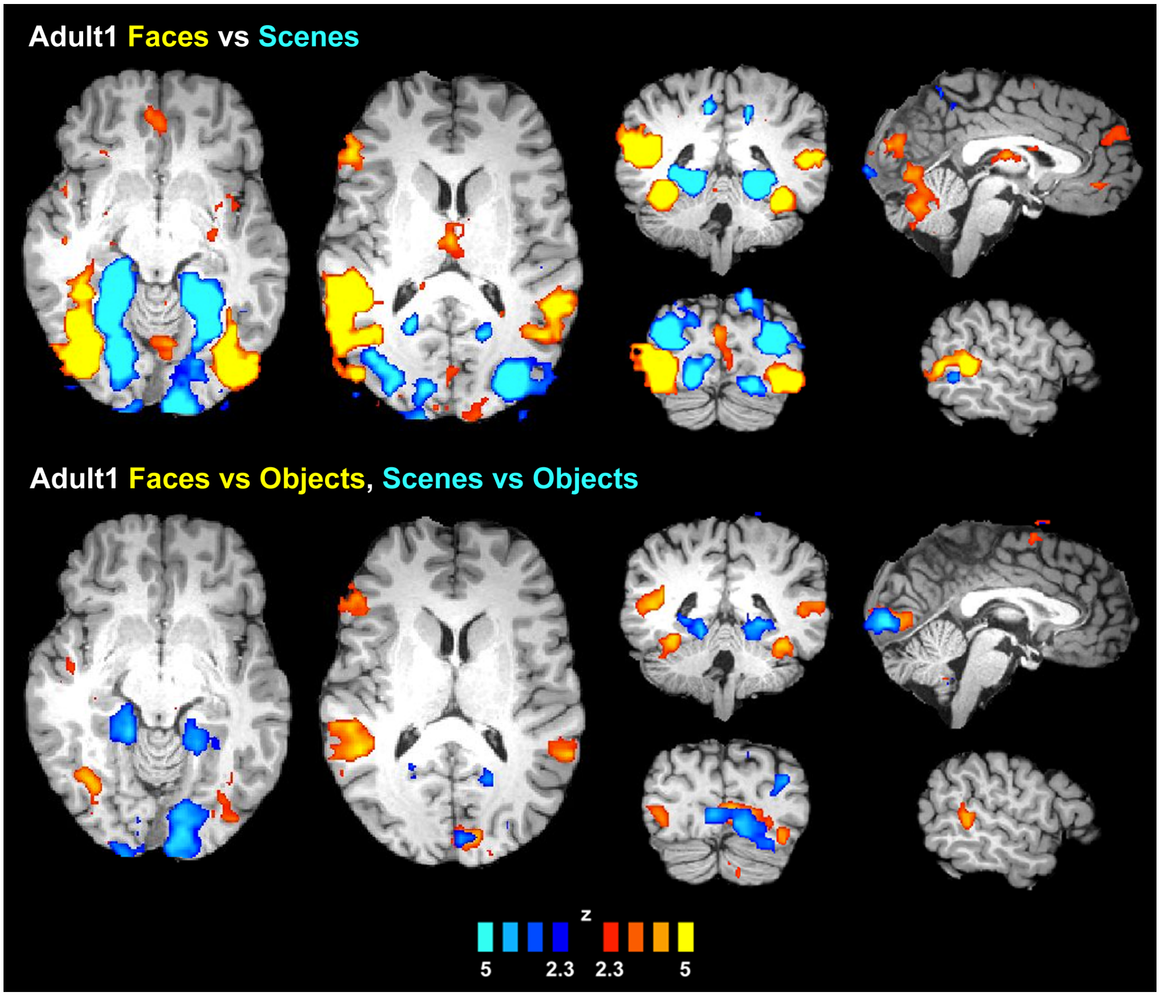

Supplementary Fig. 4: Anatomical search spaces used to define extrastriate ROIs, in infants and adults. Ventral temporal cortex includes the predicted locations of the fusiform face area and parahippocampal place area; lateral occipital cortex includes the predicted locations of the occipital face area and occipital place area; and the superior temporal sulcus (STS) includes the prediction location of the STS face area.

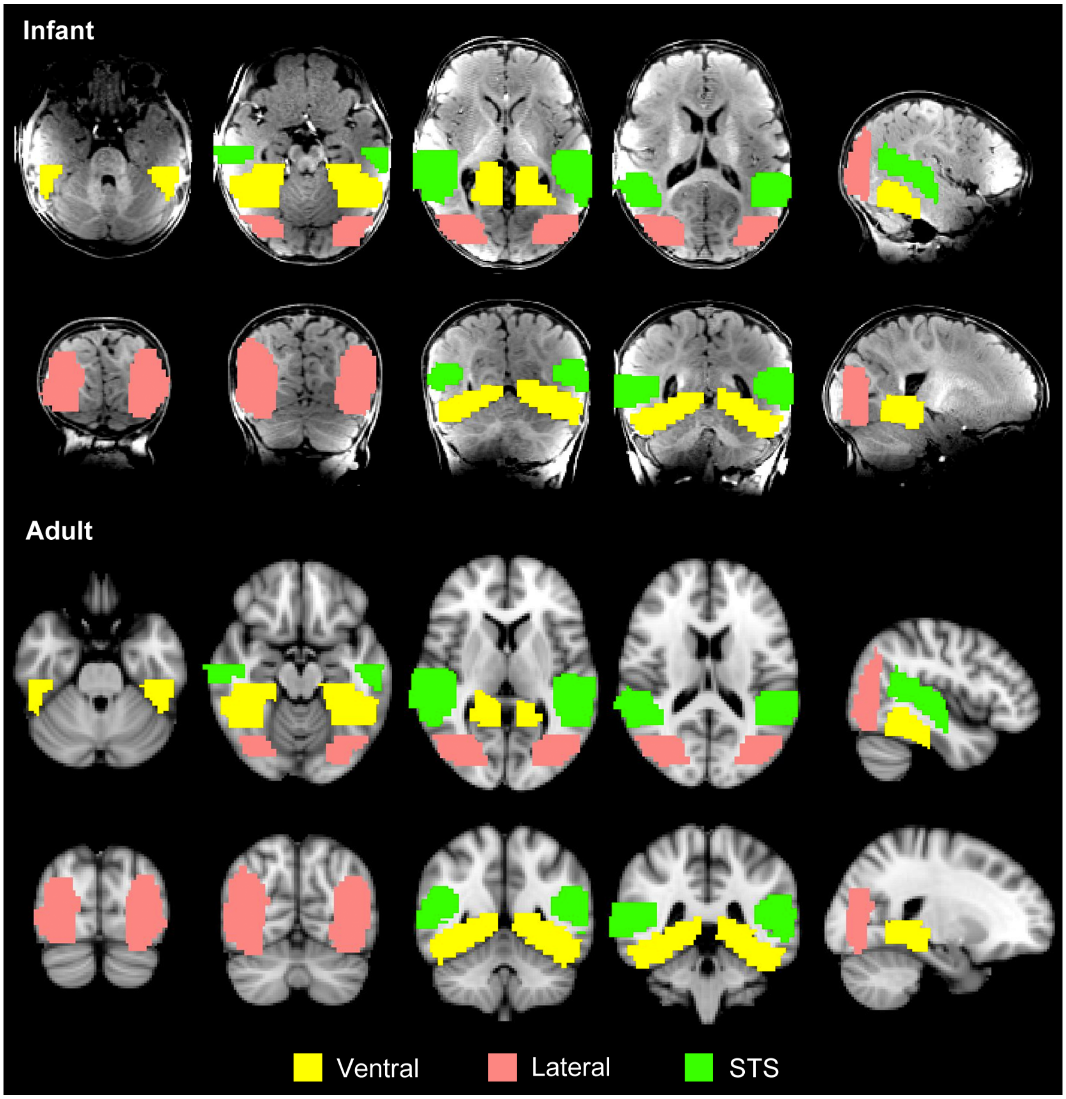

Supplementary Fig. 5: Medial prefrontal cortex (MPFC) region-of-interest (ROI) results in infants and adults. Brain images show heat maps of region-of-interest (ROI) locations across participants. Bar plots show each ROI's response (percent signal change, PSC) to faces and scenes in independent data, separately for Expt. 1 and Expt. 2-8. Error bars show the standard deviation of a permutation-based null distribution for the corresponding value. Baseline corresponds to the response to scrambled scenes (Expt. 1-3, 7-8) or scrambled objects (Expt. 4-6). Stronger responses to faces over scenes were observed in both infants (permutation test; Expt. 1, $n = 9$, $z = 2.06$, $P = .02$; Expt. 2-8, $n = 6$, $z = 1.61$, $P = .054$) and adults (permutation test; Expt. 1, $n = 3$, $z = 3.09$, $P = 1.0 \times 10^{-3}$; Expt. 2, $n = 3$, $z = 1.94$, $P = .026$).

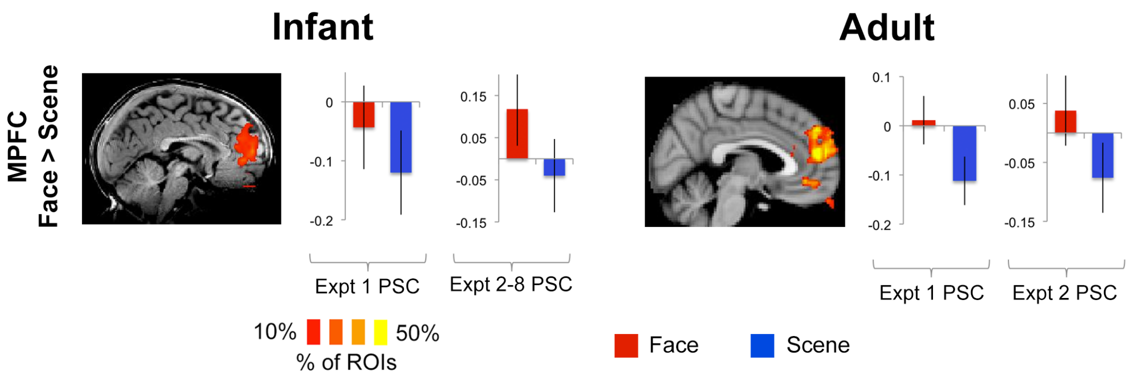

Supplementary Fig. 6: Category preferences in infants exist in both the right and left hemispheres. Brain images show heat maps of region-of-interest (ROI) locations across participants. ROIs were defined as described in the Methods section, but using separate anatomical search spaces for each hemisphere. Bar plots show each ROI's response (percent signal change, PSC) to faces and scenes in independent data, combined across Expt. 1-8. Error bars show the standard deviation of a permutation-based null distribution for the corresponding value. Baseline corresponds to the response to scrambled scenes (Expt. 1-3, 7-8) or scrambled objects (Expt. 4-6). Significant preferences for faces and scenes were observed in the right hemisphere ($n = 9$, permutation test; ventral face region, $z = 1.86$, $P = .031$; lateral face region, $z = 4.72$, $P = 1.2 \times 10^{-6}$; STS face region, $z = 5.80$, $P = 3.3 \times 10^{-9}$; ventral scene region, $z = 8.67$, $P = 2.2 \times 10^{-18}$; lateral scene region, $z = 6.48$, $P = 4.6 \times 10^{-11}$) as well as the left ($n = 9$, permutation test; ventral face region, $z = 3.76$, $P = 8.5 \times 10^{-5}$; lateral face region, $z = 3.32$, $P = 4.5 \times 10^{-4}$; STS face region, $z = 5.36$, $P = 4.2 \times 10^{-8}$; ventral scene region, $z = 8.71$, $P = 1.5 \times 10^{-18}$; lateral scene region, $z = 3.43$, $P = 3.0 \times 10^{-4}$)

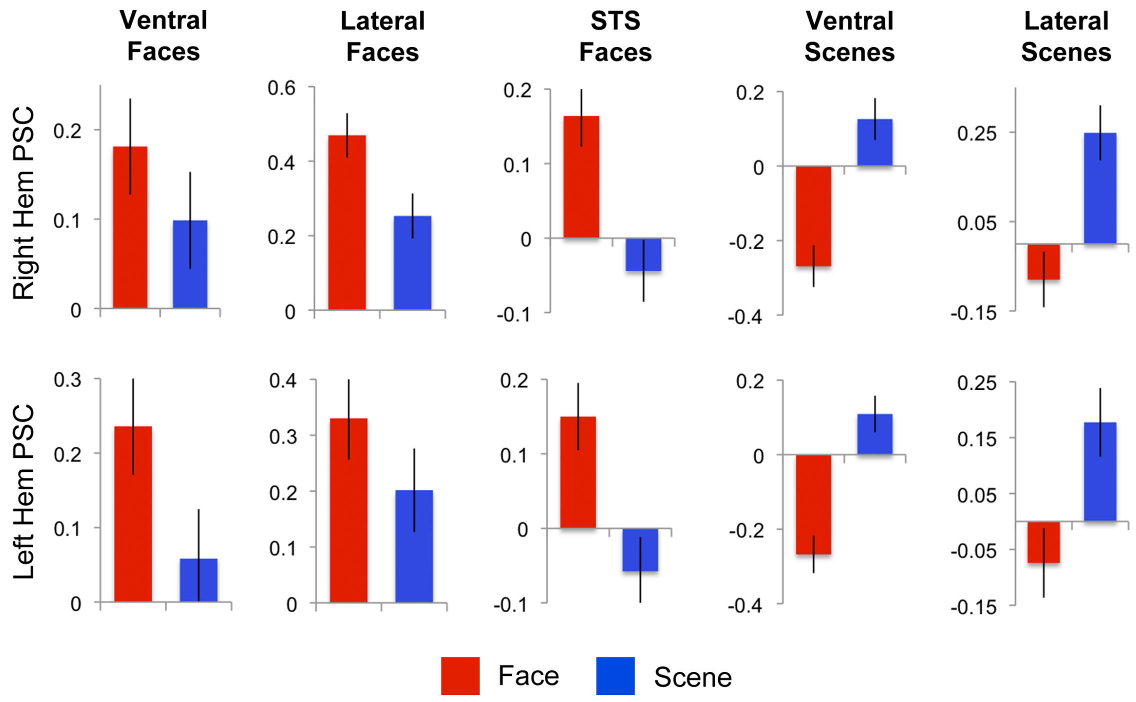

Supplementary Fig. 7: Additional region-of-interest (ROI) results, showing responses (percent signal change, PSC, in independent data) in regions defined by a face versus scene contrast in infants. **A.** Results combined across Expts. 1-8, with statistics performed across participants rather than the time-series based permutation tests used for most analyses, to explicitly show generalization across infants. Error bars show standard error of PSC values across subjects. Baseline corresponds to the response to scrambled scenes (Expts. 1-3, 7-8) or scrambled objects (Expts. 4-6). The effect of category was significant across participants in four of the five regions (n = 9; ventral face region, $t(8)$ = 2.77, P = .012; lateral face region, $t(8)$ = 3.06, $P = 7.8 \times 10^{-3}$; STS face region, $t(8)$ = 2.64, P = .015; ventral scene region, $t(8)$ = 2.91, $P = 9.8 \times 10^{-3}$), and near significant in the lateral scene region ($t(8)$ = 1.80, P = .055). **B.** Results restricted to Expt. 2, which had 80%-sized scenes and 80%-sized scrambled scenes as a baseline, to show that significant category preferences are obtained with these specific stimuli. Error bars show the standard deviation of a permutation-based null distribution for the corresponding PSC value, as in Figs. 2 and 4. The effect of category was significant for all regions (permutation test; n = 4; ventral face region, z = 2.14, P = .016; lateral face region, z = 2.91, $P = 1.8 \times 10^{-3}$; STS face region, z = 3.21, $P = 6.6 \times 10^{-4}$; ventral scene region, z = 2.35, $P = 9.4 \times 10^{-3}$; lateral scene region, z = 3.14, $P = 8.4 \times 10^{-4}$).

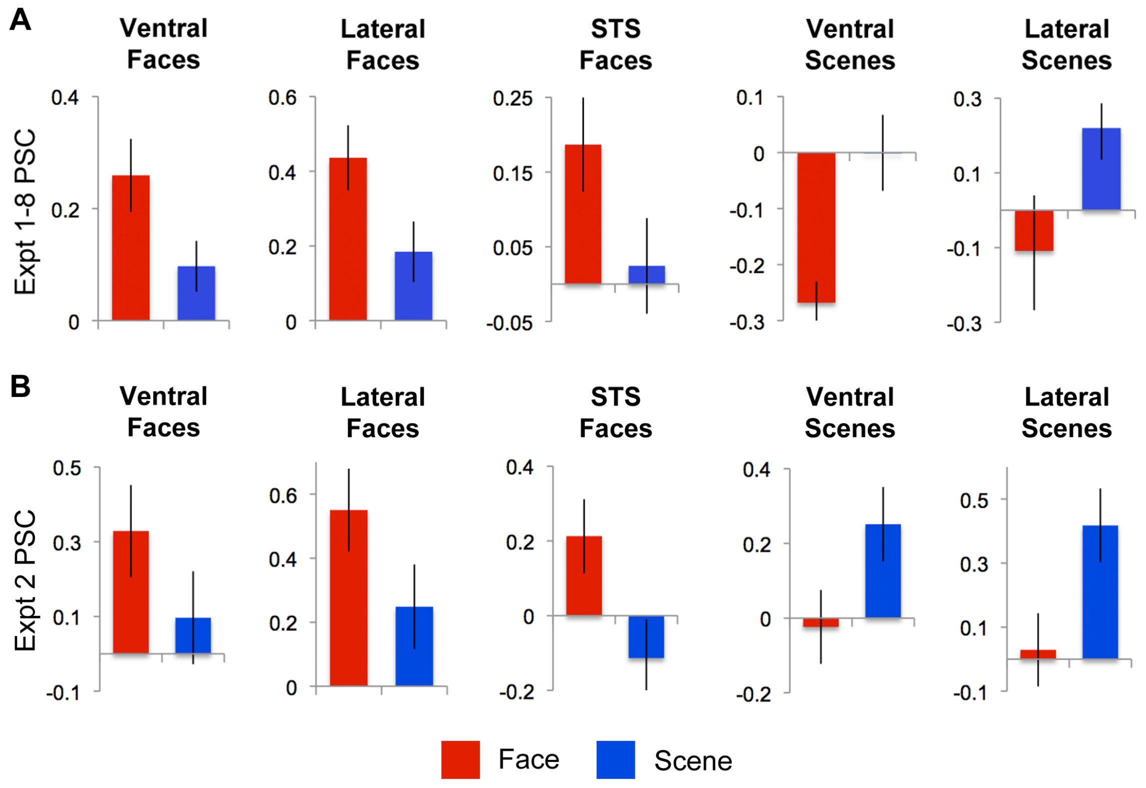

Supplementary Fig. 8: Comparison of data quality across infants and adults, for the four-condition data used to search for category-selective regions (Expt. 2-6; Fig. 4). **A.** Left: permutation-based standard error of the PSC difference of interest (faces versus objects for face regions, scenes versus objects for scene regions), in infant data, adult data, and a subset of the adult data (2/5 runs per participant). Standard errors are relatively similar across groups; in cases were adults have lower standard error, the groups are better matched by using a subset of the adult data. Right: time points included per dataset. **B.** Region-of-interest (ROI) responses (percent signal change, PSC, in independent data) in regions defined by comparing faces to objects and scenes to objects, in the subset of 2/5 runs of adult data. As with the full adult dataset, each region showed a higher response to its preferred category than to all three other categories (permutation test comparing faces or scenes to objects, $n = 3$; ventral face region, $z = 3.47$, $P = 2.6 \times 10^{-4}$; lateral face region, $z = 2.70$, $P = 3.5 \times 10^{-3}$; STS face region, $z = 4.84$, $P = 6.5 \times 10^{-7}$; ventral scene region, $z = 3.91$, $P = 4.6 \times 10^{-5}$; lateral scene region, $z = 3.07$, $P = 1.1 \times 10^{-3}$). These results suggest against the possibility that null findings in infants simply resulted from lower data quality.

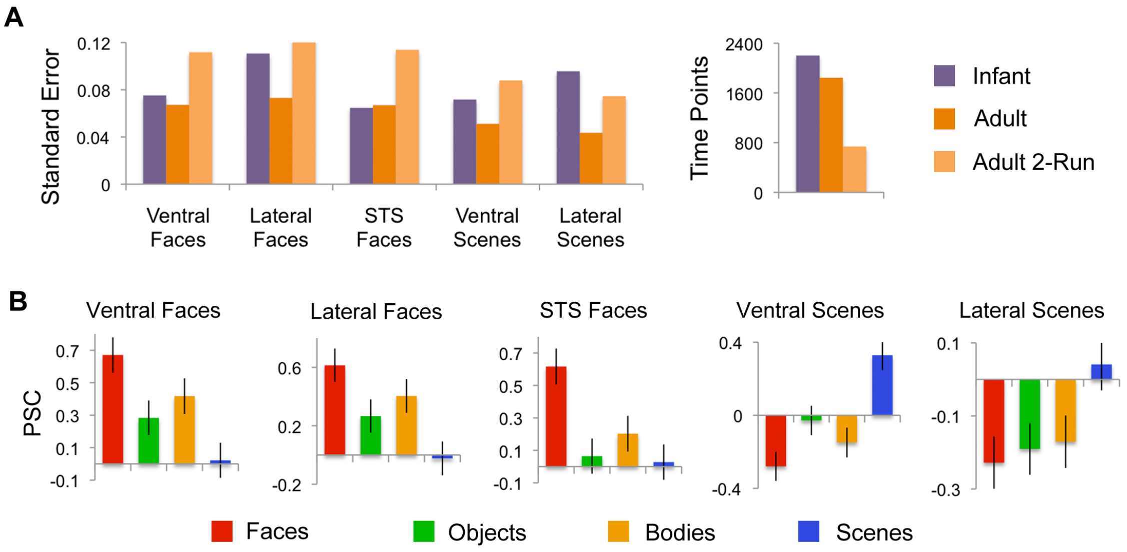

Supplementary Fig. 9: Whole-brain activation maps, comparing objects to faces (similar results were obtained comparing objects to scenes). The top two rows of images show results from the two infants with the largest amount of usable data, while the third shows results from a representative adult participant. Maps are thresholded at $P < .01$ voxelwise, and corrected for multiple comparisons using a clusterwise threshold of $P < .05$. Given the use of dynamic stimuli, these responses may reflect regions involved in analyzing the movements of objects through space, rather than a category preference for objects.

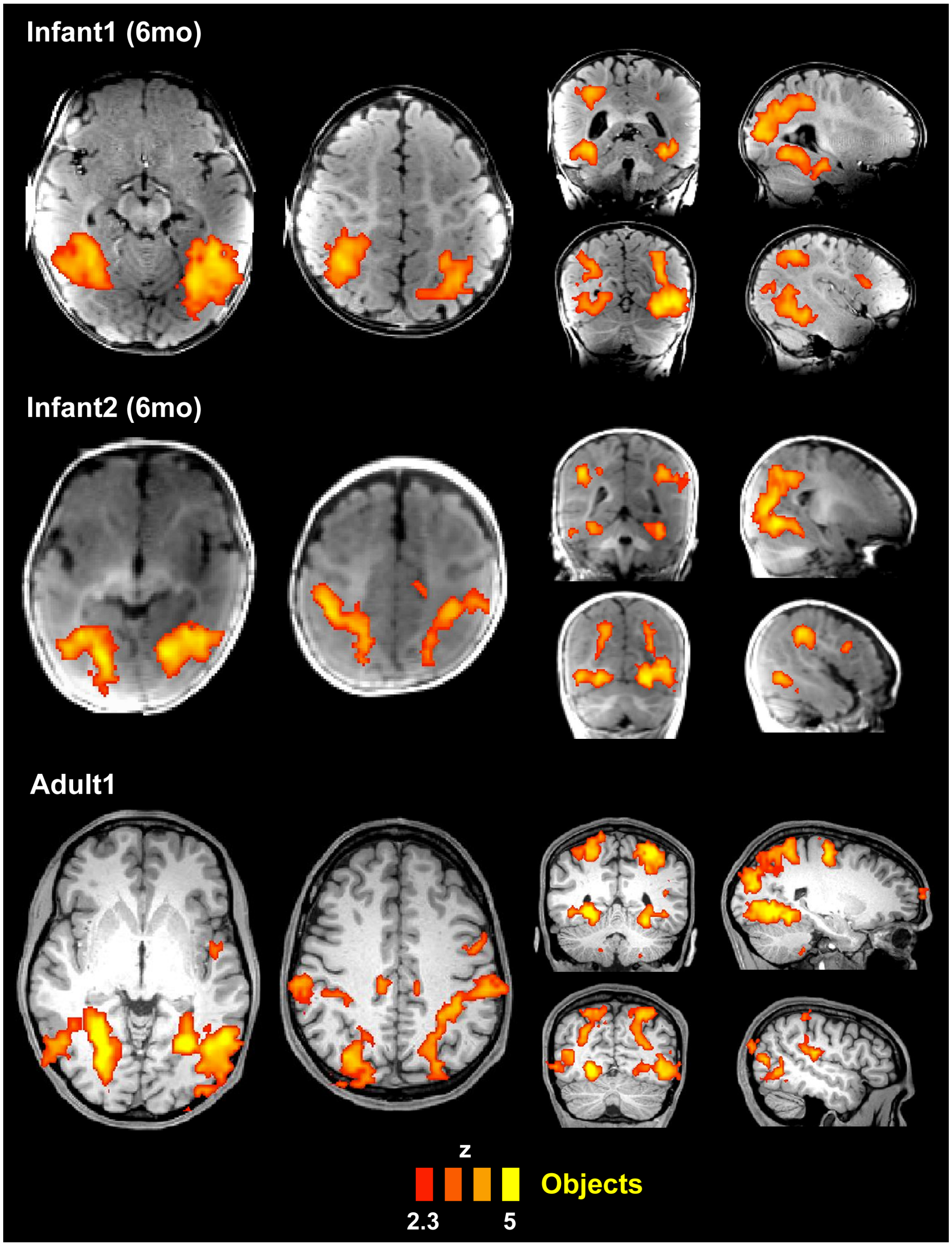

Supplementary Fig. 10: Data quantity and quality are similar across conditions in both infants and adults. Volumes kept, and mean volume-to-volume translation (mm) and rotation (degrees), from four-condition data (Expt. 2-6). This suggests that the group difference observed in representational similarity matrices (Fig. 5) is not driven by differential noise across conditions in infants versus adults.

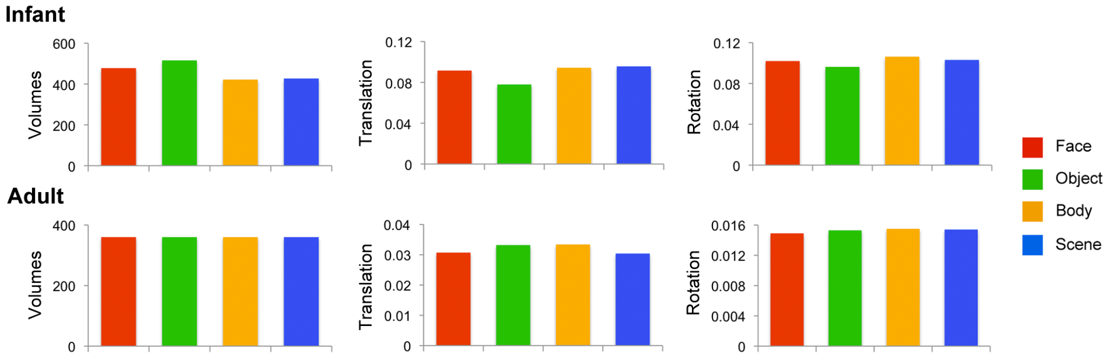

Supplementary Table 1: Participant demographic information. Many infants were scanned over multiple sessions. When sessions were separated by more than a month, they were analyzed as separate datasets. Infants are labeled by amount of data kept in their largest dataset, such that Infant1 had the largest amount of data in a single dataset.

ID	Dataset	Age (mo)	Sex	Sessions	Expts.	Participant Included	Volumes Acquired	Volumes Kept
Infant1	3/3	5.9	M	4	1, 2	1	2130	1173
Infant2	3/3	6.2	M	4	1, 2, 3	1	3140	908
Infant3	1/2	5.2	M	6	5, 6, 7	1	2748	600
Infant1	1/3	3	M	7	1	1	2626	530
Infant1	2/3	4.5	M	5	1	1	2111	479
Infant4	1/1	4.2	M	4	1, 2	1	2216	292
Infant5	1/1	7.8	M	2	1, 2	1	1136	256
Infant3	2/2	8	M	1	1, 8	1	854	234
Infant6	1/2	5.2	F	1	1	1	455	212
Infant7	2/3	4.9	M	2	1, 4	1	550	211
Infant8	1/1	4.5	M	1	1	1	209	111
Infant9	1/1	5.8	M	2	1	1	834	100
Infant6	2/2	8.6	F	1	1	0	1080	86
Infant7	3/3	6.5	M	1	4	0	320	76
Infant10	1/1	4.1	F	7	1	0	2032	31
Infant7	1/3	2.8	M	1	1	0	358	30
Infant11	1/1	3.4	M	2	1	0	820	0
Infant2	2/3	4.7	M	1	1	0	787	0
Infant2	1/3	3.3	M	1	1	0	707	0
Infant12	1/1	3.7	M	2	1	0	629	0
Infant13	1/1	2.5	M	1	1	0	566	0
Infant14	1/1	2.3	F	1	1	0	560	0
Infant15	1/1	3.9	M	2	1	0	326	0
Infant16	1/1	4.3	M	1	1	0	259	0
Infant17	1/1	4.9	M	3	1	0	222	0
Adult1	1/1	27 yrs	M	1	1, 2	1	1260	1260
Adult2	1/1	34 yrs	M	1	1, 2	1	1260	1260
Adult3	1/1	27 yrs	F	1	1, 2	1	1260	1260
